# The role of hospitals in bridging the care continuum: a systematic review of coordination of care and follow-up for adults with chronic conditions

**DOI:** 10.1186/s12913-017-2500-0

**Published:** 2017-08-09

**Authors:** Melissa De Regge, Kaat De Pourcq, Bert Meijboom, Jeroen Trybou, Eric Mortier, Kristof Eeckloo

**Affiliations:** 1 Faculty of Economics and Business Administration, Department of Innovation, Entrepreneurship, and Service Management, Ghent University, Tweekerkenstraat 2, B-9000 Ghent, Belgium; 2 Department of Strategic Policy Cell, Ghent University Hospital, De Pintelaan 185, B-9000 Ghent, Belgium; 3 Faculty of Economics, Department of Management, Tilburg University, Tilburg, The Netherlands; 4 Department Tranzo, Tilburg University, Tilburg, The Netherlands; 5 Faculty of Medicine and Health Sciences, Department of Public Health, Ghent University, Ghent, Belgium; 6 Faculty of Medicine and Health Sciences, Department of Anaesthesiology, Ghent University, Ghent University Hospital, Ghent, Belgium

**Keywords:** Chronic disease management, Hospital, Integrated care, Systematic literature review

## Abstract

**Background:**

Multiple studies have investigated the outcome of integrated care programs for chronically ill patients. However, few studies have addressed the specific role hospitals can play in the downstream collaboration for chronic disease management. Our objective here is to provide a comprehensive overview of the role of the hospitals by synthesizing the advantages and disadvantages of hospital interference in the chronic discourse for chronically ill patients found in published empirical studies.

**Method:**

Systematic literature review. Two reviewers independently investigated relevant studies using a standardized search strategy.

**Results:**

Thirty-two articles were included in the systematic review. Overall, the quality of the included studies is high. Four important themes were identified: the impact of transitional care interventions initiated from the hospital’s side, the role of specialized care settings, the comparison of inpatient and outpatient care, and the effect of chronic care coordination on the experience of patients.

**Conclusion:**

Our results show that hospitals can play an important role in transitional care interventions and the coordination of chronic care with better outcomes for the patients by taking a leading role in integrated care programs. Above that, the patient experiences are positively influenced by the coordinating role of a specialist. Specialized care settings, as components of the hospital, facilitate the coordination of the care processes. In the future, specialized care centers and primary care could play a more extensive role in care for chronic patients by collaborating.

## Background

Healthcare today is characterized by a graying population. Specifically, this trend implies larger proportions of people suffering from illnesses with a chronic course and high impact on their daily lives [1]. Beyond that, rapidly growing medical knowledge and technological innovation enables more diagnostic and treatment possibilities. Due to these trends, there is a steady increase in healthcare complexity, and coordination has become a high-priority need in healthcare systems and management [1, 2]. As chronic patients require long-term, complex healthcare responses, optimal collaboration and coordination between professionals is necessary to provide integrated and continuous care for the chronically ill [1, 2]. A major element in chronic care is the interface between hospitals, primary care providers, and community-based services [3]. A lack of coordination and integration here can cause care processes to become incoherent, redundant, and error-prone. For example, the period of discharge from hospital to home is known to be sensitive to suboptimal coordination of care, introducing concerns with respect to the quality of care [4, 5]. Hospitals will need to work closely with community partners to adequately follow-up chronic patients and to prevent avoidable hospital readmissions [6]. Forster et al. [7] reported on the frequency and severity of adverse effects following hospital discharge. Beyond this, as the applicable technology and technical knowledge grow, more services will be provided outside the hospital. Hence, hospitals will need to shift their focus from the initial role of acute care to a new additional role in chronic care.

Reducing acute hospital care for people with long-term conditions has become an important element of health policy, as governments aim to contain escalating healthcare costs [8]. Avoiding acute episodes in this group of patients is a goal in itself, and this purpose can be achieved by better chronic care. Inclusion of the acute care setting in chronic illness management is essential, because even when managed ideally, patients with chronic illnesses are frequently admitted to hospital [9]. Most experts believe that it is preferable to manage chronic disease in the ambulatory setting [9]. For example the Chronic Care Model entails changes to the health care system, mainly in the ambulatory setting, to support the development of informed, activated patients and prepared health care teams to improve outcomes [10]. In other initiatives, physician associations and employer groups have joined forces to promote the development of patient-centered medical homes in the ambulatory setting to improve the care of complex chronic illness [11, 12]. However, while advocates of outpatient chronic care argue that acute hospital care can be avoided [13], hospitals will continue to play a key role in chronic care, as most chronic conditions are characterized by acute exacerbation requiring admission. Hospitals will thus remain responsible for specific interventions [8]. Strategies need to be devised to engage hospitals and assist them in adopting innovative chronic care models not to the exclusion of, but in addition to, ambulatory care approaches. As such, hospitals will remain indispensable, but will occupy a less dominating position in the case of the chronic care patients than they employ for acute care.

Although several articles can be found regarding coordination and integrated care programs for chronically ill patients [14], too little attention has been devoted to the systematic evaluation of the current evidence for these initiatives from the perspective of hospitals and their future roles.

This article aims to examine current evidence and provide a structured, comprehensive overview of the role of hospitals in the downstream coordination and follow-up care of chronically ill patients.

The next section describes the search strategy employed, as well as the inclusion and exclusion criteria. The results are presented from four distilled perspectives of chronic disease management. The results are then integrated into the discussion, and the implications of our findings for research, practice, and policy are addressed.

## Methods

### Data sources

This study draws upon an analysis of the literature from a systematic review perspective. The Embase, Pubmed, Cinahl, EBSCO, and Web of Science databases, along with the Cochrane Library, were searched for relevant studies. The searches were conducted in March 2016. The concepts of chronic illness, integrated or transitional care, and hospitals were combined into a standardized search string using MeSH and non-MeSH entry terms:

(“delivery of health care, integrated” OR “transmural care” OR “chain care” OR “chain of care” OR “care chain” OR “care continuity, continuum of care” OR “case management” OR “disease management” OR “health network” OR “care network” OR “patient care management” OR “long term care” OR “transitional care” OR “discharge care” OR “hospital discharge” OR “coordination of care” OR “care coordination”) AND (hospitals OR “inpatient care” OR “inpatient setting” OR hospitalization) AND (“chronic disease” OR “chronic illness” OR “chronically ill” OR “chronic condition” OR comorbidity OR multimorbidity OR “multiple chronic conditions”). The initial search strategy was validated using a selection of key papers known to the authors.

### Inclusion and exclusion criteria

Our review focused on English-language papers published between 1st January 1995 and 28th February 2016. This time frame was chosen since integrated care has become an increasingly important focus of attention in healthcare literature from 1995 on [15]. In the 1990s, integrated delivery systems were set up to focus on better care coordination as a means of improving quality and reducing cost, even though most of these systems failed to deliver savings [15]. The integrated (or organized) delivery system—the first notion resembling integrated care—was described in 1994 by Shortell et al. [15, 16]. This resulted in an increased interest in academic research on integrated care, with an increasing number of publications appearing after 1995.

Only empirical quantitative and qualitative research investigating the role of hospitals in the care of chronically ill patients was included. We excluded articles unrelated to hospitals, theoretical and conceptual analysis, abstracts of meetings, review articles, editorials, and letters. Studies set in community or hospice settings, psychiatric care, or children’s care were also excluded due to the specialized nature of the settings. Finally, since studies investigating or describing individual in-hospital programs without accentuating the ‘integration’ factor cannot demonstrate the role in the continuity of care, these studies were also excluded.

### Data extraction

Two reviewers independently searched for relevant studies using the standardized search strategy described above. The selection of studies was determined through a two-step procedure. First, the search results were filtered by title and abstract, and then narrowed down according to the formal inclusion and exclusion criteria. This removed many duplicates and references to nonempirical studies. The remaining studies were selected for full-text retrieval and underwent critical quality appraisal. In the case of noncorresponding results, consensus was sought through consultation with a third reviewer. In addition, the reference lists of relevant publications were screened and a forward citation track was applied.

### Critical quality appraisal

Following Hawker et al. [17], all relevant studies were appraised using a global unweighted score based on critical appraisal to grade the accepted studies. Nine quality criteria were used and checked for every article (see Table 1). Articles with seven or more of the nine criteria were defined as high-quality studies. Studies fulfilling four, five, or six criteria were classified as medium-quality. Articles matching fewer than four criteria were described as low-quality. Each reviewer graded the empirical studies independently. Disagreements between the two raters were solved by a consensus discussion involving a third reviewer. An additional assessment of the manuscripts using an intervention on the basis of the EPOC review criteria was conducted (http://epoc.cochrane.org/epoc-specific-resources-review-authors) (Table 2).

**Table 1:** Critical quality appraisal of included articles

	Abad-Corpa et al. (2013) [18]	Akosah et al. (2002) [34]	Atienza et al. (2004) [35]	Baldwin, Black & Hammond (2014) [19]	Blue et al. (2001) [30]	Brand et al. (2004) [32]	de la Porte et al. (2007) [36]	Chiu, Shyu & Liu (2001) [38]	Cline et al. (1998) [31]	Coleman et al. (2004) [28]	Coleman et al. (2006) [29]	Cowie et al. (2009) [46]	Dossa, Bokhour & Hoenig (2012) [55]	Farrero et al. (2001) [24]	Grunfeld et al. (1999) [43]	Hanumanthu et al. (1997) [37]	Harrison et al. (2002) [23]	Ireson et al. (2009) [48]	Jeansawang et al. (2012) [27]	Ledwige et al. (2005) [22]	Linden & Butterworth (2014) [20]	Luttik et al. (2014) [41]	Moalosi et al. (2003) [39]	Naithani et al. (2006) [45]	Naylor et al. (2004) [26]	Rauh et al. (1999) [21]	Ricauda et al. (2008) [40]	Sadatsafavi et al. (2013) [42]	Shi et al. (2015) [33]	Vliet Vlietland, Breedveld & Hzaes (1997) [44]	Williams (2003)	Williams, Akroyd and Burke (2010) [25]
Quality criteria																																
Abstract and title	X	X	X	X	X	X	X	X	X	X	X	X	X	X	X	X	X	X	X	X	X	X	X	X	X	X	X	X	X	X	X	X
Background and aims	X	X	X	X			X			X		X	X		X		X	X	X	X				X	X				X		X	X
Method and data	X	X	X		X	X	X	X	X	X	X	X	X	X	X	X	X	X	X	X	X	X	X	X	X	X	X	X	X	X	X	
Sampling strategy	X	X	X	X	X	X	X	X	X	X	X	X		X	X	X	X	X	X	X	X	X	X	X	X	X	X	X	X	X	X	X
Data analysis	X	X	X		X	X	X	X	X	X	X	X	X	X	X	X	X	X	X	X	X	X	X	X	X	X	X	X	X	X	X	X
Ethical and bias	X		X			X	X		X	X	X	X		X	X		X	X	X	X	X	X	X	X	X				X		X	X
Results	X	X	X	X	X	X	X	X	X	X	X	X	X	X	X	X	X	X	X	X	X	X	X	X	X	X	X	X	X	X	X	X
Generalizability	X	X	X		X	X	X	X	X	X	X	X	X	X	X	X	X	X	X	X	X	X	X	X	X	X	X	X	X	X	X	X
Implications and usefullness	X	X	X		X	X	X	X	X	X	X	X	X	X	X	X	X	X	X	X	X	X	X	X	X	X	X	X	X	X	X	X
Score	H	H	H	M	H	H	H	H	H	H	H	H	H	H	H	H	H	H	H	H	H	H	H	H	H	H	H	H	H	H	H	H

*H* high, *M* medium

**Table 2:** Risk of bias criteria of included articles

	Abad-Corpa et al. (2013) [18]	Akosah et al. (2002) [34]	Atienza et al. (2004) [35]	Blue et al. (2001) [30]	Brand et al. (2004) [32]	de la Porte et al. (2007) [36]	Chiu, Shyu & Liu (2001) [38]	Cline et al. (1998) [31]	Coleman et al. (2004) [28]	Coleman et al. (2006) [29]	Farrero et al. (2001) [24]	Grunfeld et al. (1999) [43]	Hanumanthu et al. (1997) [37]	Harrison et al. (2002) [23]	Jeansawang et al. (2012) [27]	Ledwige et al. (2005) [22]	Linden & Butterworth (2014) [20]	Luttik et al. (2014) [41]	Moalosi et al. (2003) [39]	Naylor et al. (2004) [26]	Rauh et al. (1999) [21]	Ricauda et al. (2008) [40]	Sadatsafavi et al. (2013) [42]	Shi et al. (2015) [33]	Vliet Vlietland, Breedveld & Hzaes (1997) [44]	Williams, Akroyd and Burke (2010) [25]
Risk of bias criteria																										
Was the allocation sequence adequately generated?	High risk	High risk	Low risk	Low risk	High risk	Low risk	High risk	Low risk	Low risk	Low risk	Low risk	Unclear risk	High risk	Low risk	High risk	Unclear risk	Low risk	Low risk	High risk	Low risk	High risk	Low risk	High risk	High risk	Low risk	High risk
Was the allocation adequately concealed?	Low risk	High risk	Low risk	Low risk	Low risk	Low risk	High risk	Low risk	Low risk	Low risk	Low risk	Unclear risk	High risk	Low risk	Unclear risk	Unclear risk	Low risk	Low risk	High risk	Low risk	High risk	Low risk	High risk	High risk	Low risk	High risk
Were baseline outcome measures similar?	Low risk	Low risk	Low risk	Low risk	Low risk	Low risk	Low risk	Low risk	Low risk	Low risk	Low risk	Low risk	Low risk	Low risk	Low risk	Low risk	Low risk	Low risk	Unclear risk	Low risk	Low risk	Low risk	Low risk	Low risk	Low risk	Low risk
Were baseline characteristics similar?	Unclear risk	High risk	Low risk	Unclear risk	Low risk	Low risk	Low risk	Low risk	Low risk	Low risk	Low risk	Low risk	Low risk	Low risk	Low risk	Low risk	Low risk	Low risk	Unclear risk	Low risk	Low risk	Low risk	Low risk	High risk	Low risk	Low risk
Were incomplete outcome data adequately addressed?	Low risk	Low risk	Low risk	Low risk	Low risk	Low risk	Unclear risk	Low risk	Low risk	Low risk	Low risk	Low risk	Low risk	Low risk	Low risk	Low risk	Low risk	Low risk	Unclear risk	Low risk	Low risk	Low risk	Low risk	Unclear risk	Low risk	Low risk
Was knowledge of the allocated interventions adequately prevented during the study?	High risk	High risk	Low risk	Unclear risk	Low risk	Low risk	Low risk	Low risk	Low risk	Low risk	Low risk	Low risk	Unclear risk	Low risk	Low risk	Unclear risk	Low risk	Low risk	Low risk	Unclear risk	Low risk	Low risk	Low risk	Low risk	Low risk	Low risk
Was the study adequately protected against contamination?	Unclear risk	High risk	Low risk	High risk	Low risk	Low risk	Low risk	Low risk	Low risk	Low risk	High risk	Unclear risk	Low risk	Low risk	Unclear risk	Unclear risk	Low risk	Low risk	Low risk	Low risk	Low risk	Low risk	Low risk	Low risk	Low risk	Low risk
Was the study free from selective outcome reporting?	Low risk	Low risk	Low risk	Low risk	Low risk	Low risk	Low risk	Low risk	Low risk	Low risk	Low risk	Low risk	Low risk	Low risk	Low risk	Low risk	Low risk	Low risk	Unclear risk	Low risk	Low risk	Low risk	Low risk	Low risk	Low risk	Unclear risk
Was the study free from other risk of bias?	Low risk	Low risk	Low risk	Low risk	Low risk	Low risk	Low risk	Low risk	Low risk	Low risk	Low risk	Low risk	Low risk	Low risk	Low risk	Low risk	Low risk	Low risk	Low risk	Low risk	Low risk	Low risk	Low risk	Low risk	Low risk	Low risk

## Results

### Literature search

**Fig. 1:**
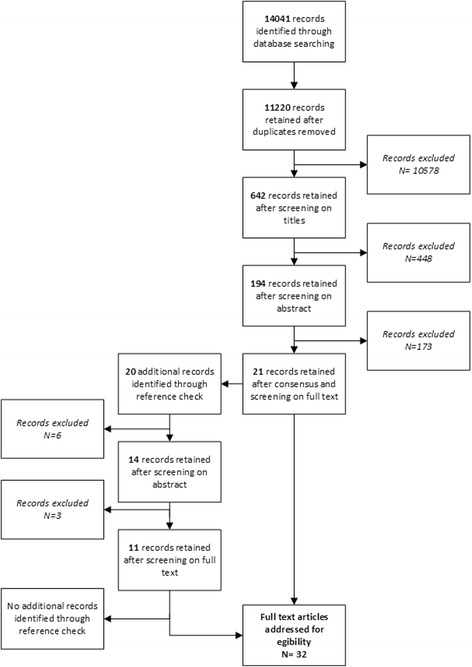
Search strategy flow diagram

Our literature search initially yielded 11,220 unique candidate articles following duplication removal (Fig. 1). Their potential relevance was examined based on their titles, and 642 were selected for abstract retrieval. On the basis of an abstract review, 448 articles were excluded from further review. After this step, the 194 references that appeared to meet the study eligibility criteria were reviewed thoroughly in full text. Several articles did not meet the inclusion criteria. Reasons for exclusion of paper in this stage where among others: not empirical research, no hospital included, not the target group, language (e.g. article just in Spanish) and systematic literature reviews. As several articles did not meet the inclusion criteria and, after consensus had been reached between the reviewers, 21 articles were included. The bibliographical references to these studies were examined to collect additional studies that had not been included in the records identified in the database search. In this way, 11 additional studies were included. As no additional studies were identified through their reference check this resulted in a final sample of 32 studies in the review.

### Quality appraisal

Table 1 summarizes the quality appraisal scores. Thirty-one studies had a score of seven or more, and can be considered high-quality papers that show a rigorous methodological approach. One paper was qualified as medium quality, which indicates good methodological rigor. Table 2 summarizes the risk of bias for intervention studies (namely randomized control trails, non-randomized controlled trails and controlled before-after studies).

### Description of studies

The studies originated from many different countries, showing the international relevance of this topic. Most were from the United States (*n* = 10) and two were from Canada. Fourteen studies are carried out in Europe (United Kingdom: *n* = 5; Spain: *n* = 3; The Netherlands: *n* = 3; Sweden: *n* = 1; Ireland: *n* = 1; and Italy: *n* = 1). Three studies were carried out in Asia, two in Australia, and one in Africa.

The selected studies differed in a number of characteristics (Table 3). First, they involved different types of patient groups: patients with heart failure, patients with diabetes mellitus, patients with rheumatoid arthritis, patients with cardiovascular disease, stroke patients, patients with chronic obstructive pulmonary diseases, and patients with chronic illnesses in general. In evaluating the results, no notable differences were found between the clinical areas. Second, several study designs can be distinguished: the majority of the studies applied a randomized control design comparing discharge and follow-up interventions with routine care for chronically ill patients. Qualitative research methods were used to examine patients’ experiences in the continuum of care. Furthermore, case studies and retrospective database analysis were employed. Third, multiple outcome measures were used, such as variables related to clinical outcomes (e.g., readmission at 30 and 90 days and 1 year; time to hospital readmission; additional hospital admissions; length of stay; mortality at 90 days and 1 year; event-free survival; emergency department presentations), determinants of the level of knowledge of the therapeutic regime (e.g., guideline adherence as well as patient adherence); quality of life (e.g., Activities of Daily Living scores), patient satisfaction and costs (e.g., average cost per patient treated). As can be seen, the universe of articles collected was quite diverse and the articles differed in methodology and intent.

**Table 3:** Findings of included studies

Reference	Country	Clinical field	Design	Outcome
Transitional care interventions				
Abad-Corpa et al. 2013 [18]	Spain	Chronic obstructive pulmonary disease (COPD)	Quasiexperimental design	Variables related to readmission, level of knowledge about the therapeutic regime, quality of life, satisfaction with nursing care
Baldwin, Black & Hammond, 2014 [19]	US	Chronic diseases	Case study	30-day readmission
Blue et al. 2001 [30]	UK	Chronic heart failure (CHF)	Randomized controlled trial	Death from all causes or hospital admission for heart failure at one year, death or hospital admission for any reason, hospital admission for worsening chronic heart failure, all-cause admission to hospital, number of patients admitted, number of admissions, days spent in hospital
Brand et al. 2004 [32]	Australia	General medical patient aged ≥ 65 years with either a history of readmissions to acute care or multiple medical comorbidities	Quasiexperimental design	Unplanned acute care readmissions (representation and hospitalization for 24 h) and emergency department presentations (discharge < 24 h after presentation) at 3 and 6 months after discharge from the index admission
Cline et al. 1998 [31]	Sweden	Heart failure (HF)	Randomized controlled trial	Hospitalization data in survivors at one year, treatment at one year follow-up
Coleman et al. 2004 [28]	US	Community-dwelling adults 65 years or older with stroke, congestive heart failure, coronary artery disease, cardiac arrhythmias, chronic obstructive pulmonary disease, diabetes mellitus, stroke, medical or surgical back conditions, hip fracture, peripheral vascular disease, or cardiac arrhythmias	Randomized controlled trial	Complicated post hospital episode; rehospitalization within 30, 90, 180 days; Emergency room (ER) or observation unit visit within 30, 90, 180 days; time to first rehospitalization, time to first ER or observation unit visit
Coleman et al. 2006 [29]	US	Community-dwelling adults 65 years or older with stroke, congestive heart failure, coronary artery disease, cardiac arrhythmias, chronic obstructive pulmonary disease, diabetes mellitus, spinal stenosis, hip fracture, peripheral vascular disease, deep venous thrombosis or pulmonary embolism	Randomized controlled trial	Rate of nonelective rehospitalization at 30, 60, 90 and 180 days after discharge; rehospitalization for same diagnosis as index hospitalization at 30, 60, 90 and 180 days after discharge
Farrero et al. 2001 [24]	Spain	COPD	Randomized controlled trial	Emergency, admissions, hospital stay
Harrison et al. 2002 [23]	Canada	HF	Randomized controlled trial	Health-related quality of life (MLHFQ, SF-36), rates of readmission, emergency room use
Jeangsawang et al. 2012 [27]	Thailand	Patients with complicated healthcare needs or a high risk of poor postdischarge outcomes	Mixed-method design	Postdischarge functional ability, health-related complications, emergency room visits, hospital readmissions, time between hospital discharge and first readmission and length of rehospitalization stays, family member satisfaction
Ledwidge et al. 2005 [22]	Ireland	HF	Randomized controlled trial	Mortality rates, HF-related admission rate
Linden & Butterworth, 2014 [20]	US	HF and COPD	Randomized controlled trial	30 and 90 day readmission incidence rates, ER visits incidence rates, mortality rates
Naylor et al. 2004 [26]	US	HF	Randomized controlled trial	Time to first rehospitalization or death, number of rehospitalization, quality of life, functional status, costs, and satisfaction with care
Rauh et al. 1999 [21]	US	CHF	Retrospective analysis	Length of stay, admission and readmission rates, costs to the patient and provider
Williams et al. 2010 [25]	UK	CHF	Quasiexperimental design	Readmission, length of stay, patient satisfaction
Specialized care setting				
Akosah et al. 2002 [34]	US	CHF	Chart review	Hospital readmissions at 30 and 90 days and 1 year; risk reduction; time to hospital readmission, additional hospital admissions; mortality at 90 days and 1 year; event free survival
Atienza et al. 2004 [35]	Spain	High-risk of HF	Randomized controlled trial	Rate of events per observation year, rate of readmitted patients per observation year, for CHF, not for CHF, rate of readmissions per observation year, for CHF, not for CHF, rate of deaths per observation year
de la Porte et al. 2007 [36]	The Netherlands	HF	Randomized controlled trial	Hospitalization for CHF or death, death (all causes), days in hospital
Hanumanthu et al. 1997 [37]	US	HF	Retrospective analysis	Hospitalization rate, exercise capacity
Hospital versus nonhospital care				
Chiu et al. 2001 [38]	Taiwan	Stroke patients with severe physical disabilities	Case-control study	Changes in Activities of Daily Living scores (ADL) scores, family costs for caregiving
Grunfeld et al. 1999 [43]	UK	Breast cancer	Randomized controlled trial	Patient satisfaction
Luttik et al. 2014 [41]	The Netherlands	CHF	Case-control study	Guideline adherence, patient adherence, number of deaths, hospital readmission, unplanned hospital readmissions
Moalosi et al. 2003 [39]	Africa	Chronically ill tuberculosis patients	Case-control study	Outcome (died during treatment, completed treatment, defaulted, transferred out), average cost per patient treated
Ricauda et al. 2008 [40]	Italy	COPD	Randomized controlled trial	Mortality, readmission to hospital, length of stay, depression, quality of life, cost per patient per day
Sadatsafavi et al. 2013 [42]	Canada	Asthma	Chart review	Direct asthma-related medical cost, rate of readmission, asthma related outpatient service use, rate of short-acting β-agonist dispensation, days covered with a controller medication
Shi et al. 2015 [33]	China	Hypertension or diabetes	Case-control study	Quality and value of care, access, continuity, coordination, comprehensiveness, satisfaction, cost concerns, and health improvement
Vliet Vlieland et al. 1997 [44]	The Netherlands	Rheumatoid arthritis	Randomized controlled trial	Swollen and tender joint counts, patients’ assessment of pain, patients’ and the physicians’ assessments of disease activity, ESR, HAQ
Experiences and expectations of patients				
Cowie et al. 2009 [46]	UK	Arthritis, coronary heart disease, stroke, hypercholesterolemia, hypertension, diabetes mellitus, or chronic obstructive pulmonary disease	Qualitative interview study	Longitudinal and relational continuity, management continuity between organizations, access and flexibility
Dossa et al. 2012 [55]	US	70 years or older, two or more chronic conditions or mobility impairments	Qualitative longitudinal interview study	Communication
Ireson et al. 2009 [48]	US	CHF, diabetes, COPD, colon cancer, or breast cancer	Qualitative interview study	Preparation for specialist visit, patient’s experience during the specialist visit, trust of physician
Naithani et al. 2006 [45]	UK	Diabetes	Qualitative interview study	Experienced longitudinal continuity, experienced flexible continuity, experienced team and crossboundary continuity
Williams 2004 [47]	Australia	Multiple chronic conditions	Qualitative phenomenological study	Perceptions of quality of care of acute care services

By performing content analysis of the studies, four different themes (perspectives) emerged from the articles. For the analysis phase all the selected articles where read through making a descriptive evaluation of the literature. Notes were made to mark relevant information in the papers. Data was fractured and analyzed directly, initially through open coding for the emergence of a core category. Consequently, different items were categorized. The author identified whether or not the categories could be linked any way and listed them in four major themes. Finally, two researchers independently allocated the articles to the different groups. In the case of noncorresponding results, consensus was sought through consultation with a third reviewer.

The majority of the articles (15, 47%) described a transitional care intervention originating from a hospital to enhance the discharge and follow-up process for chronically ill patients. Closely related was the perspective of specialized settings providing care after hospital discharge; this was studied in four (12%) articles. A third perspective, found in eight (25%) articles, involved looking at outcomes in hospital care versus nonhospital care for chronically ill patients. The final perspective was the experiences and expectations of chronically ill patients towards the continuity of their illness during or after hospitalization (5, 16%). The results in each of these dimensions will be described separately (see also Tables 3 and 4).

**Table 4:** Methodological overview of included studies

Reference	Purpose	Intervention	Control group	Findings
Transitional care interventions				
Abad-Corpa et al. 2013 [18]	To evaluate effectiveness of protocoled intervention for hospital discharge and follow-up in primary care	At the hospital, a coordinating nurse visited each patient in the experimental group to identify the main caregiver, provide information about the disease, explain treatment, identify care problems and needs, and facilitate communication between professionals. Twenty-four hours after discharge, the coordinating nurses informed the primary care nurses of patient discharge. The two nurses made the first home visit together. There were follow-up phone calls at 2, 6, 12, and 24 weeks after discharge. Number of included participants *n* = 56.	In the control group, no extra plan was carried out and the specific healthcare protocol of each hospitalization unit was followed. Number of included participants *n* = 87.	Improvement in quality of life at 12 and 24 weeks after discharge; level of knowledge of Chronic Obstructive Pulmonary Disease (COPD) revealed significant differences between the groups. No differences according to satisfaction or readmission rate. The intervention was ineffective in reducing readmission rates.
Baldwin, Black & Hammond, 2014 [19]	To develop a community nursing case management (CMM) program to decrease preventable readmissions to the hospital and emergency department by providing telephonic case management and, where needed, onsite assessment and treatment by a clinical nurse specialist (CNS) with prescriptive authority	The patients were identified by CNS census review or referral from clinical nurse leaders, case managers, emergency department physicians, or staff, based on the program inclusion criteria. Once identified, the CNS meets with the patient during inpatient stay and discusses participation in the outpatient program. The CCM program consists of telephone appointments on a weekly basis, or more frequently, based on patient acuity, for a period of 30 days after discharge (goal). Number of included participants *n* = 39.	No control group.	A positive change in hospital culture since the program began. The CNS is receiving more referrals. There is also more dialogue between the CNS and hospitalists regarding the plan of care for patients who will be entering the CCM program after discharge. Participating patients interact with the CNS and have expressed gratitude for the program. Hospital and ED 30-day readmissions for program patients have decreased since the program began.
Blue et al. 2001 [30]	To determine whether specialist nurse interventions improve outcome in patients with chronic heart failure	A specialist nurse led intervention starting at the hospital. The intervention consisted of a number of planned home visits of decreasing frequency, supplemented by telephone contact as needed. The aim was to educate the patient about heart failure and its treatment, optimize treatment (drugs, diet, exercise), monitor electrolyte concentrations, teach self-monitoring and management (especially the early detection and treatment of decompensation), liaise with other health care and social workers as required, and provide psychological support. Number of included participants *n* = 84.	Patients in the usual care group were managed as usual by the admitting physician and, subsequently, general practitioner. They were not seen by the specialist nurses after hospital discharge. Number of included participants *n* = 81.	31 patients (37%) in the intervention group died or were readmitted with heart failure compared with 45 (53%) in the usual care group. Compared with usual care, patients in the intervention group had fewer readmissions for any reason (86 vs. 114, *P* = 0.018), fewer admissions for heart failure (19 vs. 45, *P* < 0.001) and spent fewer days in hospital for heart failure (mean 3.43 vs. 7.46 days, *P* = 0.0051).
Brand et al. 2004 [32]	To determine whether a nurse-led chronic disease management model of transitional care reduces readmissions to acute care	The intervention group received a comprehensive transitional care service with components allocated according to perceived and assessed need and patient preference. The patient was seen by the chronic disease nurse consultant in the 24 h before discharge from the ward and the following were completed: collection of predischarge data; screening for risk factors for readmission; development of a plan for follow-up in clinic; liaison with discharge planners, nursing staff and allied health staff, where appropriate; provision of an action plan for the patient; copy of discharge summary faxed to the patient’s general practitioner. The patient was seen again by the nurse in the chronic disease center within two weeks of discharge (or when the medical condition allowed). Number of included participants *n* = 76.	Usual care. Number of included participants *n* = 78.	There was no difference between the control and intervention groups in readmission rates or emergency department presentation rates at the three-month and six-month follow-ups. Scores for quality of life showed no difference between groups at three-month follow-up. There was no difference between the groups in the rate of visits to a general practitioner for the three months following their index admission. The findings from the qualitative process evaluation identified major issues that impacted on the effectiveness and sustainability of the transitional care service model: inadequate integration of the chronic disease nurse consultant into the existing general medical delivery of care model; inadequate stakeholder understanding of the role and scope of the chronic disease nurse consultant inadequate clerical support resources for the chronic disease nurse consultant; and lack of backfill for leave coverage, such that a 52-week-per-year service can be provided; inadequate integration of documentation into daily clinical practice.
Cline et al. 1998 [31]	To study the effects of a management program on hospitalization and health care costs one year after admission for heart failure	A management program for heart failure starting at the hospital. The intervention group received education on heart failure and self-management, with follow up at an easy access, nurse-directed outpatient clinic for one year after discharge. Number of included participants *n* = 56.	The control group was managed according to routine clinical practice. Number of included participants *n* = 79.	The mean time to readmission was longer in the intervention group than in the control group and the number of days in hospital tended to be fewer. There was a trend towards a mean annual reduction in health care costs per patient in the intervention group compared with costs in the controls.
Coleman et al. 2004 [28]	To test whether an intervention designed to encourage older patients and their caregivers to assert a more active role during care transition can reduce rehospitalization rates	The intervention was built on four pillars, or conceptual domains: (1) assistance with medication self-management, (2) a patient-centered record owned and maintained by the patient to facilitate cross-site information transfer, (3) timely follow-up with primary or specialty care, and (4) a list of red flags indicative of a worsening condition and instructions on how to respond to them. The four pillars were operationalized through the following two mechanisms: (1) a personal health record and (2) a series of visits and telephone calls with a transition coach. Number of included participants *n* = 158.	Usual care. Number of included participants *n* = 1235.	There was a significant difference in favor of the rehospitalization of intervention subjects with that of controls at 30 days, at 90 days, and at 180 days. Intervention patients reported high levels of confidence in obtaining essential information for managing their condition, communicating with members of the healthcare team, and understanding their medication regimen.
Coleman et al. 2006 [29]	To test whether a care transition intervention reduces rehospitalization rates	The intervention was built on four pillars, or conceptual domains: (1) assistance with medication self-management, (2) a patient-centered record owned and maintained by the patient to facilitate cross-site information transfer, (3) timely follow-up with primary or specialty care, and (4) a list of red flags indicative of a worsening condition and instructions on how to respond to them. The four pillars were operationalized through the following two mechanisms: (1) a personal health record and (2) a series of visits and telephone calls with a transition coach. Number of included participants *n* = 379.	Usual care. Number of included participants *n* = 371.	Intervention patients had lower rehospitalization rates at 30 days and at 90 days than control subjects. Intervention patients had lower rehospitalization rates for the same condition that precipitated the index hospitalization at 90 days and at 180 days than the controls. The mean hospital costs were lower for intervention patients vs. controls at 180 days.
Farrero et al. 2001 [24]	To analyze the influence of a hospital-based home-care program (HCP) on the management of patients with COPD receiving long-term oxygen therapy	The HCP applied to patients in the intervention group (HCP group) consisted of a monthly telephone call, home visits every three months, and home or hospital visits on a demand basis. Number of included participants *n* = 60.	Patients in the control group were given conventional medical care. Number of included participants *n* = 62.	During the follow-up period, there was a highly significant decrease in the mean number of emergency department visits also a significant decrease in hospital admissions and days of hospital stay in the HCP group. Cost analysis showed a total saving of 8.1 million pesetas ($46,823) in the HCP group, mainly due to a decrease in the use of hospital resources. There was no difference in pulmonary function, gas exchange, quality of life, or survival between the two groups.
Harrison et al. 2002 [23]	Evaluate whether the use of usual providers, and a reorganization of discharge planning and transition care with improved intersectoral linkages between nurses, could improve quality of life and utilization of health services for individuals admitted to hospital with heart failure	The nurse-led intervention focused on the transition from hospital-to-home and supportive care for self-management two weeks after hospital discharge. Number of included participants *n* = 92.	Usual care. Number of included participants *n* = 100.	Six weeks after hospital discharge, the overall Minnesota Living with Heart Failure Questionnaire (MLHFQ) score was better among the transitional care patients than among the usual care patients. Similar results were found at twelve weeks after discharge for the overall MLHFQ and at six and twelve weeks after discharge for the MLHFQ’s Physical Dimension and Emotional Dimension subscales. Differences in generic quality life, as assessed by the SF-36 Physical component, Mental Component, and General Health subscales, were not significantly different between the transition and usual care groups. At twelve weeks after discharge, 31% of the usual care patients had been readmitted compared with 23% of the transitional care patients, and 46% of the usual care group had visited the emergency department, compared with 29% in the transitional care group.
Jeangsawang et al. 2012 [27]	To compare the outcomes of discharge planning and follow-up care, for elders with chronic healthcare conditions, among advanced practice nurse (APN), expert-by-experience nurses, and novice nurses who delivered care through a “Continuity of Care Program”, and to describe the benefits of APN care services from the perspectives of key stakeholders (e.g., healthcare colleagues and family caregivers)	Continuity of Care Program: The discharge planning and postdischarge follow-up care was offered through the hospital ambulatory care unit. The program consisted of care services aimed at maximizing the patient’s health and functionability by addressing existing problems and preventing potential problems. The delivery of services involved the use of three levels of practitioners (novice, expert-by-experience, and advanced practice nurses) who functioned as primary home healthcare nurses. All three types of nurses used the same standard of care, addressed in the Continuity of Care Program, to guide their nursing interventions. Nursing interventions for discharge planning and postdischarge follow-up consisted of: preparing the patient and his/her family caregiver to be ready for the discharge; coordinating all aspects of the discharge and postdischarge follow-up plan; conducting a series of home visits to assess and monitor the caregiving ability of the family caregiver, and identify the presence of health-related complications and implement appropriate treatments; and, providing care management support to the family caregiver. These services began once a discharge consultation was requested by a physician and while the patient was still hospitalized. Number of included participants APN = 20; expert-by-experience nurses *n* = 40; Novice nurse *n* = 40.		Only family members’ satisfaction with the nursing care their elder received was significantly different between the three groups. Although family caregivers in all three groups rated the quality of discharge planning and follow-up care as highly satisfactory, satisfaction with the APN care was significantly higher than satisfaction score with the expert-by-experience nurse and novice nurse care. The APN was seen as a useful healthcare provider in a complex healthcare system. Three themes emerged from the data: a) provision of comprehensive care for older patients with complex healthcare problems; b) professional interactions with patients and other members of the healthcare team; c) professional collaboration with the physician.
Ledwidge et al. 2005 [22]	To examine the additional benefits of extending the standard three-month heart failure (HF) program to six months on death and readmission over a two-year follow-up period	Patients randomized to the HF program received weekly telephone calls from one of three experienced specialist HF nurses who, in most cases, was the specialist nurse who had managed the patient during the first three months following discharge. The purpose of these unscripted telephone calls was to determine clinical stability of the patient, address any questions or concerns they had and revise key education points as deemed necessary by the nurse. The key education points revised concerned daily weight monitoring, disease and medication understanding, compliance with prescribed therapy, and the dietary salt restriction. An in-house educational book about HF was supplied and used in the clinic and phone education sessions with patients and carers. This book served to standardize the education points provided to patients by nurses and covered the disease, its causes, associated symptoms and investigations, and pharmacological and nonpharmacological treatments. Patients were asked to attend the HF clinic at six and twelve weeks after randomization for formal clinical assessment by clinic medical and nursing staff. This included history and physical examination by a physician, review of HF medications and key education issues by the nurse, and a nutrition review by the dietician. Number of included participants *n* = 62.	Usual care. Number of included participants *n* = 68.	There was no measured clinical advantage in terms of death or HF readmission in extending a structured hospital-based disease management program for HF beyond three months after discharge. However, it appears that patients continue to need access to the service to help avoid clinical deterioration, and this may have implication for the optimal organization of such programs.
Linden & Butterworth, 2014 [20]	To inform the development of successful transitional care interventions	The intervention included a comprehensive set of components: 1) predischarge components: patient education, discharge planning; medication reconciliation, scheduling of follow-up appointment; 2) postdischarge components: timely follow-up, follow-up telephone call, availability of patient hotline; and 3) bridging components: transition/health coach, patient-centered discharge instructions. Two postdischarge components were added-motivational interviewing-based health coaching and symptom monitoring using interactive voice responses. Number of included participants *n* = 253.	Usual care. Number of included participants *n* = 259.	There was no statistical difference between treatment groups on 30-day readmission incidence rates or 90-day readmission incidence rates. Groups also did not differ in Emergency Department visit incidence rates at 30 or 90 days. The mortality rate among patients with Chronic Heart Failure (CHF) showed no difference between groups. However, COPD mortality at 90 days was lower in the intervention group than in the usual care group.
Naylor et al. 2004 [26]	To examine the effectiveness of a transitional care intervention delivered by APNs to elders hospitalized with heart failure	The intervention included all of the following components: (1) a standardized orientation and training program guided by a multidisciplinary team of heart failure experts to prepare APNs to address the unique needs of older adults and their caregivers throughout an acute episode of heart failure; (2) use of care management strategies foundational to the Quality–Cost Model of APN transitional care; and (3) APN implementation of an evidence-based protocol, guided by national heart failure guidelines and designed specifically for this patient group and their caregivers with a unique focus on comprehensive management of needs and therapies associated with an acute episode of heart failure complicated by multiple comorbid conditions. The protocol consisted of an initial APN visit within 24 h of index hospital admission, APN visits at least daily during the index hospitalization, at least eight APN home visits (one within 24 h of discharge), weekly visits during the first month (with one of these visits coinciding with the initial follow-up visit to the patient’s physician), bimonthly visits during the second and third months, additional APN visits based on patients’ needs, and APN telephone availability seven days a week. Number of included participants *n* = 118.	Control group patients received care routine for the admitting hospital including site-specific heart failure patient management and discharge planning critical paths and if referred, standard home agency care consisting of comprehensive skilled home health services seven days a week. Standards of care for all study hospitals include institutional policies to guide, document, and evaluate discharge planning. The discharge planning process across hospital sites was similar. The attending physician was responsible for determining the discharge date, and the primary nurse, discharge planner, and physician collaborated on the design and implementation of the discharge plan. Standards and processes of care for the primary home care sites were also similar. These included use of liaison nurses to facilitate referrals to home care; availability of comprehensive, intermittent skilled home care services in patients’ residences seven days a week; and on-call registered nurse availability 24 h per day. Number of included participants *n* = 121.	Time to first readmission or death was longer in intervention patients. At 52 weeks, patients in the intervention group had fewer readmissions and lower mean total costs. For intervention patients, only short-term improvements were demonstrated in overall quality of life, the physical dimension of quality of life, and patient satisfaction.
Rauh et al. 1999 [21]	An analysis of patients with a primary diagnosis of congestive heart failure at discharge, before and after the implementation of a comprehensive inpatient and outpatient congestive heart failure program consistent with the guidelines of the Agency for Health Care Policy and Research.	A comprehensive inpatient and outpatient congestive heart failure program consisting of an intensive education program focusing on diet, compliance and symptom recognition, as well as outpatient infusions. It also incorporated aggressive pharmacological treatment for patients with advanced congestive heart failure. Number of included participants *n* = 347	Historical comparative data of patients who received regular HF care. Number of included participants *n* = 407.	Decreases in length of stay, admission and readmission rates, costs to the patient and provider.
Williams et al. 2010 [25]	To evaluate the effectiveness of a transitional care service on readmissions and length of stay in hospital for patients with CHF	The transitional care intervention required the Clinical nurse specialist (CNS) to identify and recruit patients within 24–48 h of admission. The heart failure CNS visited this group of patients regularly on the wards throughout their admission, during which time they received information on their heart condition in preparation for discharge. The conversation was nurse-led, but was an interactive discussion between the patient and the nurse. The development of the intervention was based on facilitating the transition of the CHF patient from hospital to home. Therefore, follow-up arrangements either involved attendance at the nurse-led clinic or, where appropriate, home visits by the community heart failure nurse. Number of included participants *n* = 47.	Historical comparative data of patients who received regular HF care. Number of included participants *n* = 50.	The number of readmissions was higher in the control group compared with the transitional care group. Difference in length of stay for both groups almost achieved statistical significance. Patients gave positive feedback about the service.
Specialized care settings				
Akosah et al. 2002 [34]	To determine if disease management in a short-term, aggressive-intervention heart failure clinic (HFC) following hospital discharge is associated with improved outcomes	A heart failure clinic was designed as a multispecialty, short-term management program for patients with heart failure. The core management team is composed of three cardiologists with expertise in CHF, two nurse practitioners, and a nurse educator. The team interacts closely with other disciplines; including nephrology, pulmonology, and endocrinology to provide for easy access to reciprocal consultation services, such as a nutritionist and exercise physiologist. A plan of care is developed at enrolment. During this time, the patient’s medications are aggressively titrated, requiring frequent monitoring of laboratory values and physical examination. With intensive education, patients are taught self-care skills in the management of their disease. Timing of follow-up visits is flexible and individualized to each patient based on their response to therapy. Number of included participants *n* = 38.	Patients were put in the control group if they had not been referred to the heart failure clinic, but had received follow-up care within the usual care system, and had left ventricular systolic dysfunction as the basis for their CHF. Number of included participants *n* = 63.	There was a trend toward shorter times to the first outpatient visit following discharge, more outpatient visits within 90 days, and more patient-initiated contacts in the heart failure clinic group than in the control group. The combined hospital readmission and mortality rate at 90 days and a year was lower in the heart failure clinic group. There was a 77% relative risk reduction for 30-day hospital readmission in favor of heart failure clinic group, and a statistically lower rate of readmission at 90 days and a year. Utilization and maintenance of standardized CHF medications were significantly higher in patients who attended the heart failure clinic.
Atienza et al. 2004 [35]	Assess the effectiveness of a discharge and outpatient management program in a nonselected cohort of patients hospitalized for HF	The intervention program consisted of three phases: 1) the patients and their family received formal education about their disease; 2) a visit to the primary care physician was scheduled within two weeks of discharge to monitor the patient’s clinical progress, identify incipient physical signs of decompensation, and reinforce knowledge; 3) regular follow-up visits to the outpatient heart failure clinic were scheduled every three months where, in addition to the routine clinical assessments, the cardiologist reviewed the patients’ performance and introduced corrective strategies to improve treatment adherence and response. The heart failure specialist coordinated visits to other specialists, diagnostic test and treatments prescribed by other instances. A telemonitoring phase lasted from discharge until the end of the study. Number of included participants *n* = 164.	Control patients received discharge planning according to routine protocol of study hospitals. Number of included participants *n* = 174.	After a median of 509 days, there were fewer events (readmission or death) in the intervention than in the control group (156 vs. 250), which represents a 47% event reduction per observation year. At one year, time to first event, time to first all-cause and HF readmission, and time to death had increased in the intervention group. All-cause and HF readmission rates per observation year were significantly lower, quality of life improved, and overall cost of care was reduced in the intervention group.
de la Porte et al. 2007 [36]	To determine whether an intensive intervention at a heart failure (HF) clinic using a combination of a clinician and a cardiovascular nurse, both trained in HF, reduces the incidence of hospitalization for worsening HF or all-cause mortality (primary end point) and improves functional status (including left ventricular ejection fraction, New York Heart Association (NYHA) class, and quality of life) in patients with NYHA class III or IV	The intervention consisted of 9 scheduled patient contacts—at day 3 by telephone, and at weeks 1, 3, 5, 7 and at months 3, 6, 9 and 12 by a visit—with a combined, intensive physician-and-nurse-directed HF outpatient clinic, starting within a week after hospital discharge from the hospital or referral from the outpatient clinic. Verbal and written comprehensive education, optimization of treatment, easy access to the clinic, recommendations for exercise and rest, and advice for symptom monitoring and self-care were provided. Number of included participants *n* = 118.	Usual care.	The number of admissions for worsening HF or all-cause deaths in the intervention group was lower than in the control group. There was an improvement in left ventricular ejection fraction in the intervention group compared with the usual care group. Patients in the intervention group were hospitalized for a total of 359 days compared with 644 days for those in the usual care group. Beneficial effects were also observed on NYHA classification, prescription of spironolactone, maximally reached dose of β-blockers, quality of life, self-care behavior, and healthcare costs. Number of included participants *n* = 122.
Hanumanthu et al. 1997 [37]	To determine whether hospitalization rates and functional outcomes are improved when patients are managed by physicians with special expertise in heart failure working in a dedicated heart failure program	Patients were followed on a long-term basis by three physicians who work exclusively with heart failure and heart transplantation patients. Two nurse coordinators assisted with the management of patients both during hospitalizations and as outpatients. Home health agencies were involved in the care of ≈10% of the patients. All patients referred to the program underwent echocardiographic evaluation and, whenever possible, cardiopulmonary exercise testing. Exercise testing is typically repeated at three to six months to monitor patient status, if the patient is able to exercise. Exercise testing with concurrent hemodynamic monitoring is used extensively in the evaluation and management of patients, as described previously. Program offices are based in a single area in the Vanderbilt outpatient office building. Cardiopulmonary exercise testing and exercise hemodynamic testing are performed by program staff in an outpatient laboratory adjacent to the office area. Number of included participants *n* = 134.		In the year before referral, 94% of patients were hospitalized (210 cardiovascular hospitalizations), compared with 44% of the patients during the year after referral (104 hospitalizations) (53% reduction). Heart failure decreased from 164 to 60 for all patients regardless of follow-up duration and decreased from 97 to 30 (69% reduction) for patients followed up at least a year after referral. Loop diuretic doses were on averaged doubled during the first six months after referral.
Hospital versus nonhospital care				
Chiu et al. 2001 [38]	To compare the cost and effectiveness of long-term institutional care and home care	Number of included participants: Patients receiving care in a chronic care unit in a hospital *n* = 106; patients admitted to nursing home *n* = 60; patients receiving professional home nursing care *n* = 60; patients returned home without receiving professional care *n* = 80		Caring for patients in their own homes was not only more expensive but was also less effective in improving ADL scores than caring for patients in nursing homes and in hospital chronic care units.
Grunfeld et al. 1999 [43]	To assess the effect on patient satisfaction of transferring primary responsibility for following up with women with breast cancer in remission from hospital outpatient clinics to general practice	Routine follow-up in hospital outpatient clinics. Number of included participants *n* = 138.	Routine follow-up with their own general practitioner, primary care. Number of included participants *n* = 122.	The general practice group selected responses indicating greater satisfaction than did the hospital group on virtually every question. Furthermore, in the general practice group, there was a significant increase in satisfaction over baseline; a similar significant increase in satisfaction over baseline was not found in the hospital group.
Luttik et al. 2014 [41]	The aim of the current study was to determine whether long-term follow-up and treatment in primary care is equally effective as follow-up at a specialized heart failure clinic in terms of guideline adherence and patient adherence	HF clinic; number of included participants *n* = 92	Primary care; Number of included participants *n* = 97	After twelve months, no differences between guideline adherence, as estimated by the Guideline Adherence Indicator, and patient adherence, were found between treatment groups. There was no difference in the number of deaths, and hospital readmissions for cardiovascular reasons were also similar. The total number of unplanned non- cardiovascular hospital readmissions, however, tended to be higher in the primary care group than in the HF clinic group.
Moalosi et al. 2003 [39]	To determine the affordability and cost effectiveness of home-based directly observed therapy compared to hospital-based for chronically ill tuberculosis patients, and to describe the characteristics of patients and their caregivers	Home based directly observed therapy for chronically ill tuberculosis patients. Number of included participants patients *n* = 50.	Hospital based directly observed therapy for chronically ill tuberculosis patients. Number of included participants (other TB patients) *n* = 56.	Overall, home-based care reduced the cost per patient treated by 44% compared with hospital-based treatment. The cost to the caregiver was reduced by 23%, while the cost to the health system was reduced by 50%. Home-based care is more affordable and cost-effective than hospital-based care for chronically ill TB patients, although costs to caregivers remain high in relation to their incomes.
Ricauda et al. 2008 [40]	To evaluate hospital readmission rates and mortality at a six-month follow-up in selected elderly patients with acute exacerbation of COPD	Inpatient care in a general medical ward. Number of included participants *n* = 52.	Geriatric home hospitalization service. Number of included participants *n* = 52.	There was a lower incidence of hospital readmissions for Geriatric home hospitalization service patients than for general medical ward patients at six- month follow-up (42% vs. 87%). Cumulative mortality at six months was 20.2% in the total sample, without significant differences between the two study groups. Patients managed in the Geriatric home hospitalization service had a longer mean length of stay than those cared for in the general medical ward. Only Geriatric home hospitalization service patients experienced improvements in depression and quality-of-life scores. On a cost per patient per day basis, Geriatric home hospitalization service costs were lower than costs in general medical ward.
Sadatsafavi et al. 2013 [42]	To examine if the secondary care provided by specialists following episodes of asthma-related hospitalization is associated with better outcomes compared with the primary care provided by general practitioners	Secondary care group. Number of included participants *n* = 1044.	Primary care group. Number of included participants *n* = 1044.	There was no difference in the direct asthma-related costs and rate of readmission between the secondary and the primary care groups. Patients under secondary care had a higher rate of asthma-related outpatient service use, but a lower rate of short acting β-agonist dispensation. The proportion of days covered with a controller medication was higher among the secondary care group. Compared with those who received only primary care, patients who received secondary care showed evidence of more appropriate treatment. Nevertheless, there were no differences in the costs or the risk of readmission. Adherence to asthma medication in both groups was poor, indicating the need to raise the quality of the care provided by generalists and specialists alike.
Shi et al. 2015 [33]	To examine the impact of an integrated care delivery intervention on health care seeking and outcomes for chronically ill patients	Interval counties with an integrated care delivery model for promoting appropriate health care utilization by improving access and coordination through the adoption of computerized clinical pathways, a shift from fee-for-service to case-based payment, performance-based payment for care providers, and information technology-based monitoring on service quality of health care facilities. Comparison between rural health stations and hospital users. Number of included participants *n* = 199	Control counties, comparison between rural health stations and hospital users. Number of included participants *n* = 172	Significant associations between types of health care facilities as well as value of care were observed in favor of the rural health stations.
Vliet Vlieland et al. 1997 [44]	To evaluate whether the improvement achieved by a short inpatient multidisciplinary treatment program could be maintained over a 1-year period	Inpatient treatment consisted of primary medical and nursing care, daily exercise therapy, occupational therapy, and support from a social worker. Treatment goals and modalities were discussed during weekly multidisciplinary team conferences. During outpatient care, the prescription of medication and paramedical treatment was left to the attending rheumatologist at the outpatient clinic in both groups. Number of included participants *n* = 39.	Treatment regimens normally employed in the outpatient clinic. Number of included participants *n* = 41.	Averaged over the time points 2, 52, and 104 weeks, the improvement was significantly greater in the inpatient group than in the outpatient group, except for the erythrocyte sedimentation rate and Health Assessment Questionnaire score.
Experiences and expectations of patients				
Cowie et al. 2009 [46]	To examine patients’ experiences of continuity of care in the context of different long-term conditions and models of care, and to explore implications for the future organization care of long-term conditions	Number of included participants *n* = 33.		Multiple morbidity was frequent and experiences of continuity were framed within patients’ wider experiences of health care rather than the context of a particular diagnosis. Positive experiences of relational continuity were strongly associated with long-term general practitioner-led or specialist-led care. Management continuity was experienced in the context of shared care in terms of transitions between professionals or organizations. Access and flexibility issues were identified as important barriers or facilitators of continuity.
Dossa et al. 2012 [55]	Describe patient and caregiver experiences with care transitions following hospital discharge to home for patients with mobility impairments receiving physical and occupational therapy	Number of included participants *n* = 18, nine patients, nine caregivers.		Breakdowns in communication in four domains impacted continuity of care and patient recovery: (a) poor communication between patients and providers regarding on-going care at home, (b) lack of clarity about who to contact after discharge, (c) Provider response to phone calls following discharge, and (d) provider–provider communication.
Ireson et al. 2009 [48]	To examine the quality of the information received by patients with a chronic condition from the referring and specialist physician in the specialist referral process and the relationship between the quality of information received and trust in physicians	Number of included participants *n* = 250.		Most patients (85%) received good explanations of the reason for the specialist visit from the referring physician, yet 26% felt unprepared about what to expect. Trust in the referring physician was highly associated with the preparatory information patients received. Specialists gave good explanations of diagnosis and treatment, but 26% of patients received no information about follow-ups. Trust in the specialist correlated highly with good explanations of diagnosis, treatment, and self-management.
Naithani et al. 2006 [45]	To identify patients’ experiences and values with respect to continuity in diabetes care	Number of included participants *n* = 25.		Patients’ accounts identified aspects of care they valued that were consistent with four dimensions of experienced continuity of care. These were receiving regular reviews with clinical testing and provision of advice over time (longitudinal continuity); having a relationship with a usual care provider who knew and understood them, was concerned and interested, and took time to listen and explain (relational continuity); flexibility of service provision in response to changing needs or situations (flexible continuity); and consistency and co-ordination between different members of staff, and between hospital and general practice or community settings (team and crossboundary continuity). Problems regarding a lack of experienced continuity mainly occurred at the transitions between sites of care, between providers, or with major changes in patients’ needs.
Williams, 2004 [47]	To investigate perceptions of quality of care by patients experiencing comorbidities who required an acute hospital stay	Number of included participants *n* = 12.		Data analysis revealed three themes: poor continuity of care for comorbidities, the inevitability of something going wrong during acute care and chronic conditions persisting after discharge. Combinations of chronic illnesses and treatments affected these patients’ experiences of acute care and recovery after discharge. Medicalized conceptualizations of comorbidity failed to capture the underlying health care needs of these patients.

### Transitional care interventions

A total of 15 papers evaluated the effectiveness of transitional care interventions initiated within the hospital. The interventions consisted of comprehensive transitional care interventions with different steps. Patients were identified during their inpatient stay and followed up during and after discharge. These follow-ups were coordinated by transition coaches (such as specialized nurses and case managers). Besides the follow-up, most interventions included varying assistance, such as medication self-management, patient-centered records, red flags indicative of the patient’s condition, and education programs and access to outpatient clinics for the patients after hospitalization [18–33]. All but one [19] compared the interventions with control groups of patients receiving the usual care.

Several authors demonstrated lower readmission rates for the intervention patients than for control subjects [21, 23–26, 28–30] and lower hospital costs [24, 29, 31]. This in contrast with Abad-Corpa et al. [18], Brand et al. [32], Cline et al. [31], Linden & Butterworth [20], and Ledwidge et al. [22], who found no difference between the control and intervention groups in readmission rates. Other cited positive outcomes for the intervention patients included high levels of confidence in managing their condition and understanding their medical regimen [28], significant improvements in quality of life after discharge [18, 26], and patient satisfaction [25, 26]. However, Farrero et al. [24] and Adab-Corpa et al. [18] could not confirm this higher patient satisfaction.

On organizational level, Baldwin et al. [19] described a positive change in hospital culture since the beginning of the transitional care program (e.g., more dialogue between healthcare providers). However, Brand et al. [32] identified major issues (such as patient factors and local system issues like inadequate integration of the program, inadequate stakeholder understanding of the program, inadequate clerical support resources, and inadequate integration of documentation) that have an impact on the effectiveness and sustainability of the transitional care model.

Jeangsawang et al. [27] compared the effect of transitional care programs between three different type of nurses—namely, advanced practice nurses (APNs), expert-by-experience nurse, and novice nurses. Only the satisfaction of family members in favor of the APNs was significant. The APNs were seen as useful healthcare providers in a complex healthcare system.

### Specialized care settings

Three studies examined the effect of interventions at a heart failure clinic compared to usual care [34–36] (Table 4). In these studies, a heart failure clinic was designed, as a multiple specialty, short-term management program for patients with heart failure, implying comprehensive hospital discharge planning and close follow-up at these heart clinics after hospital discharge. These heart clinics are thus components of the hospital. Overall, the results for such clinics showed positive effects in terms of lower hospitalization duration, fewer hospital readmissions, lower mortality rates, and improvement in clinical outcomes (e.g., left ventricular ejection fraction) [34–36]. The quality of life improved and the cost of care were reduced in the intervention group [35, 36]. Similar results were found in the study of Hanumanthu et al. [37]. They examined whether a heart failure program managed by physicians with expertise in heart failure could improve hospitalization rates and financial outcomes; they found positive effects in terms of reductions in hospitalization after initiation of the program.

### Hospital care versus nonhospital care

Our review identified three articles that compared the effectiveness of long-term institutional care versus home-based care (Table 4). The findings were mixed; on one hand, Ciu et al. [38] stated that caring for patients in their own homes was more expensive and less effective. On the other hand, Moalosi et al. [39] found that home-based care is more affordable and reduced costs, while Ricauda et al. [40] found a lower incidence of hospital readmissions and shorter length of stay for Chronic Obstructive Pulmonary Disease (COPD) geriatric patients in geriatric home hospitalization wards than for patients at general medical wards.

Additionally, four papers studied follow-ups for chronically ill patients in secondary versus primary care (Table 4). The results of Luttik et al. [41] showed that the number of readmissions tended to be higher in the primary care group than in the heart failure clinic group; Sadatsafavi et al. [42] found that patients in secondary care showed evidence of more appropriate treatment; however, they could not demonstrate reductions in cost or readmissions. However, patient satisfaction was higher for patients in follow-ups for cancer care with their general practitioner than in hospital outpatient clinics [38, 43]. Shi et al. [33], found that hospitals did not provide a higher quality of care in terms of coordination of medication, referrals, and services received, compared to rural health stations.

Finally, one paper evaluated the improvement achieved by a short inpatient treatment program for rheumatoid arthritis versus outpatient care [44], and showed a significantly greater improvement in clinical outcomes for the inpatient group than for the outpatient group [44].

### Experiences and expectations of patients

Some other important variables identified in five of the articles are the patients’ experiences and values with respect to the continuity of care in the context of long-term conditions (Table 4). Naithani et al. [45] described four dimensions of continuity of care experienced in diabetes: (1) longitudinal continuity (receiving regular reviews with clinical testing and advice over time), (2) relational continuity (having a relationship with one care provider who knew and understood the patient, was concerned and interested, and who took the time to listen and explain), (3) flexible continuity (flexibility of service provision in response to changing needs or situations), and (4) team and crossboundary continuity (consistency and coordination between different members of staff and between hospital and general practice or community settings). The study revealed that most problems occurred at transition points; thus, with a lack of crossboundary continuity between sites or between providers or a lack of flexibility in coordination when there are major changes in the patient’s needs. Cowie et al. [46] showed that relational continuity was positively correlated with long-term specialist-led care, illustrating that patients need continuity; this can even originate from a hospital (i.e., specialist-led care). They also demonstrated that access to care and flexibility issues were important barriers and facilitators of continuity. Investigating the perceptions of quality of care by chronically ill patients who require acute hospital stays, Williams [47] revealed three themes: (1) patients perceive poor continuity of care, especially for comorbidities, (2) it is inevitable that something goes wrong during acute care, and (3) chronic conditions persist after discharge. The combinations of chronic illness and treatment affected the patients’ experiences of acute care and recovery following discharge. Ireson et al. [48] looked at the quality of information received by patients and the relationship between this information and trust in the physician. Most patients received good explanations for the reason for a specialist visit, but felt unprepared about what to expect. Beyond that, specialists give good explanations of diagnosis and treatment, but not about follow-ups to treatment. Trust in the specialist correlated highly with good explanations of diagnosis, treatment, and self-management [48].

## Discussion

In care delivery models (such as the Chronic Care model) the importance of the hospital in chronic illness management is recognized [9]. This also holds for the fact that attending to acute illness episodes is integral to the delivery of chronic illness care. As such, including elements from the hospital sector in chronic illness management is essential. This paper provides an overview of the empirical literature on the role of hospitals in chronic disease management. Our aim was to synthesize the available, somewhat fragmentary, evidence. This study outlines different types of clinical fields, diverse methodologies, and multiple outcome measures. The results are structured following four large domains: the impact of transitional care interventions, the role of specialized care settings, the comparison of inpatient and outpatient care, and the effect of chronic care coordination on the experience of patients. The type of integrated care interventions and the effects varied across the different studies; however, some important insights follow from the published results.

Most of the integrated care research focused on the outcome of integrated care programs. These integrated care programs seem to have positive effects on the quality of care. However, there are widely varying definitions and components of integrated care programs [15], while the specific role of the hospitals is often neglected. Most of the integrated care programs in our systematic review, which thus focused on the role of the hospital, included structured clinical follow-ups and case management, often combined with self-management support and patient education. A large number of the articles show that these integrated care programs originating from the hospital have positive effects; like the reduction of hospital readmission [21, 23–26, 28–30] and lower costs [24, 29]. Note, however, that we did not include studies with integrated care programs originating from outside the hospitals, so we cannot compare these programs.

However, there are also articles demonstrating that not all integrated care interventions are successful [18, 20, 22, 31, 32] and that there are impeding factors, such as the difficulty of implementing integrated care programs [32], thus showing the complexity of integrated care for chronically ill patients. This has also been described by Cramm et al. [49] who showed that the implementation of transition programs requires a supportive and stimulating team climate to enhance the quality of care delivery to chronically ill adolescents.

The transition of care for the chronically ill also impacts patient perceptions [25, 26]. The coordinating role of a specialist influences the patient experience in a positive way [19, 27]. Specialists input -to diagnosis, initial assessment, and treatment- is essential. A chronic condition may well have large implications, and specialist expertise ensures optimum treatment and offers the best chance of maintaining health. As such, hospitals can be an entry and follow up point for the chronically ill patient.

Continuity of care is very important. This finding supports the necessity for more research on hand-overs in healthcare processes [50]. Other studies show the importance of case managers [51] and patient care teams [52] in transitional care interventions. In this literature review, we did not investigate who is required to take the lead in the coordination of care for the chronically ill. However, different roles are observed for hospitals. Hospitals play an important role in the coordination of transitional processes, and our results show that this coordination can be managed by case managers (such as advanced nurses) from within the hospitals; the role of a specialized case manager or coordination program was identified as highly important by the patients [37, 46]. As a result, hospitals should be organized into process-oriented teams (physicians and nurses) and seek to coordinate integrated care for chronically ill.

General practitioners were also identified as playing coordinating roles [43, 46]. However, it seems that primary care is perceived by the patient as less efficient and of lower quality than secondary care, above that, specialized care settings provide better results compared to primary care [41, 42]. But, as we saw, primary care can also be important in integrated care programs [33, 43]. As such, an increase in integrated care arrangements might introduce a shift of some tasks guided by hospitals to either primary care or more specialized care services. Hospital units with a focus on specific pathologies might not only break the current boundaries of medical departments but also challenge the boundaries between the different healthcare partners. Such ‘vertical networks’ (collaborations between organizations with different service offerings) can improve coordination and thus service delivery for the chronically ill [53]. Further research on this topic, mainly on how this collaboration can be organized, is recommended.

To the best of our knowledge, our study is the first comprehensive attempt to evaluate the role of the hospital for patients with chronic illness. However, the study has several potential limitations. The most obvious is the relatively small sample size of articles evaluating the specific role of hospitals in chronic disease management. Longitudinal studies constitute an important avenue for future research. Beyond that, some articles could have been missed, as we specifically targeted those looking at the role of hospitals in chronic disease management, rather than in chronic disease management in general. We did not focus on studies solely studying elderly or pediatric patients, as in these groups different actors are involved than in the regular adult care. However, studies focusing on elderly are extremely important since the role of the hospital in the coordination of care and follow-up for elderly might be considerable. Hence, further research in the domain elderly care is recommended. Above that, the results are based on a limited number of search terms and as MeSH terms were used, some papers could have been excluded from the results as the process of indexing papers is not immediate.

Additionally, the review did not capture gray literature, publically available literature not published in peer reviewed journals, and thus not all relevant articles may have been included. Another limitation of the study is that the heterogeneous nature of the studies (in terms of interventions, patient population, types of outcomes, and settings) and the methodological deficiencies identified did not permit the use of formal statistical techniques, such as meta-analysis [54]. Meta-analysis makes it possible to correct for random errors, though not for systematic errors or influencing factors, such as study setting or patient population. Therefore, good descriptions of the studies and interpretation of the results, as provided in our review, are still necessary. Caution should be employed in generalizing the conclusions of our review.

## Conclusion

In the view of the changing healthcare context and the dehospitalization of care, we have addressed an important topic. Hospitals play an important role in transitional care interventions and in the coordination of care. Specialized care settings also invest in the coordination of these processes. In the future, specialized care centers and primary care will play a more extensive role in the care for chronic patients and will have to collaborate.

## Abbreviations

ADL: Activities of Daily Living Scores
APN: Advanced practice nurses
CHF: Chronic Heart Failure
CMM: Community Nursing Care
CNS: Clinical Nurse Specialist
COPD: Chronic Obstructive Pulmonary Disease
ER: Emergency room
H: High
HCP: Home-care program
HF: Heart failure
M: Medium
MLHFQ: Minnesota Living with Heart Failure Questionnaire
NYHA: New York Heart Association
UK: United Kingdom
US: United States
